# The transformation of isolated gastric myeloid sarcoma into acute myeloid leukemia presenting with a complex karyotype and TLS-ERG gene fusion: A case report

**DOI:** 10.1097/MD.0000000000029475

**Published:** 2022-05-27

**Authors:** Lu Gao, Yan Xu, Zuguo Tian, Junxiu Xia, Zhong Yuan, Di Chen, Mingqiang Ren

**Affiliations:** a Department of Hematology, Affiliated Hospital of Zunyi Medical University, Zunyi 563000, Guizhou Province, China; b School of Nursing, Medicine & Technology, College of Zunyi Medical University, Zunyi 563003, Guizhou Province, China; c Department of Nursing, Zigong Fourth People's Hospital, Zigong, 643099, Sichuan Province, China.

**Keywords:** abdominal pain, acute myeloblastic leukemia, complex karyotype, myeloid sarcoma, TLS-ERG

## Abstract

**Rationale:**

Isolated myeloid sarcoma (MS) is characterized by the rapid proliferation of myeloblasts of acute myeloid leukemia (AML), without any blood or bone marrow involvement. This disease can manifest with extramedullary organ involvement, such as the skin, lymph nodes, bone, brain, breast cervix, and visceral organs, while the occurrence of myeloid sarcomas in the stomach is rare. Isolated MS has been associated with acute myeloid leukemia (AML), but the rapid progression of MS to acute myeloid leukemia with a complex karyotype and TLS-ERG fusion gene is even rarer.

**Patient concerns:**

A 33-year-old woman suffered from persistent epigastric pain accompanied by two months of anorexia and nausea, as well as 1-week of melena.

**Diagnosis:**

This patient was initially diagnosed with gastric MS that eventually transformed into AML with a complex karyotype and TLS-ERG fusion gene, 4 months later.

**Interventions:**

Only palliative care, including nutrition support, antacids, blood transfusion, anti-infection methods were used on this patient to determine the cachexia status and the family's requirement.

**Outcomes:**

Routine follow-up results demonstrated this patient had died due to cerebral hemorrhage five months after the diagnosis of MS.

**Lessons:**

Comprehensive integration of patient history, imaging features, mass and bone marrow biopsy, and molecular cytogenetic may provide insights that could help us avoid the misdiagnosis of gastric MS. Isolated gastric MS can rapidly progress to AML with a poor prognosis if the patient does not receive appropriate treatment.

## 1 Introduction

Myeloid sarcoma (MS) is a rare extramedullary neoplasm that consists of immature myeloid cells.^[1]^ It is characterized by a highly variable outcome and usually disrupts the normal tissue architecture at its site of origin. MS can occur de novo or concurrent with other hematological malignancies, such as acute myeloid leukemia (AML), myeloproliferative neoplasm, or myelodysplastic syndrome.^[2]^ Isolated MS, also known as primary or de novo MS occurs without bone marrow or other extramedullary site involvement at the time of diagnosis and accounts for 1%–2% of MS.^[3]^ According to 2016 revision World Health Organization classification of myeloid neoplasms and acute leukemia, MS is recognized as a subtype of AML.^[4]^ Existing clinical evidence indicates that isolated MS frequently occurs in the skin, lymph nodes, bone, brain, breast, cervix, and visceral organs. In this report, we present the rare case of 33-year-old women who was diagnosed with isolated gastric MS that rapidly progressed to AML with a complex karyotype and TLS-ERG fusion gene, four months later.

## 2 Case presentation

**Figure 1:**
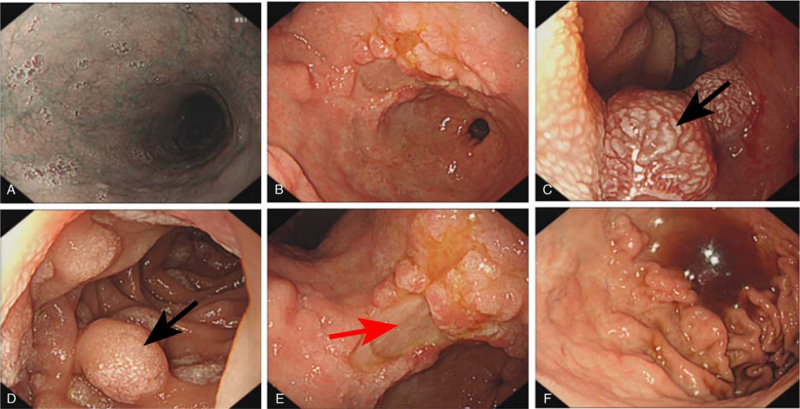
Gastroscopy examination of the morphology of the esophagus (A), gastric antrum (B), pylorus (C), descending duodenum (D), gastric angle (E), and gastric body (F). Note: Ulcers at the gastric angle and multiple nodular projections in the pylorus and descending duodenum are highlighted using red and black arrows, respectively.

**Figure 2:**
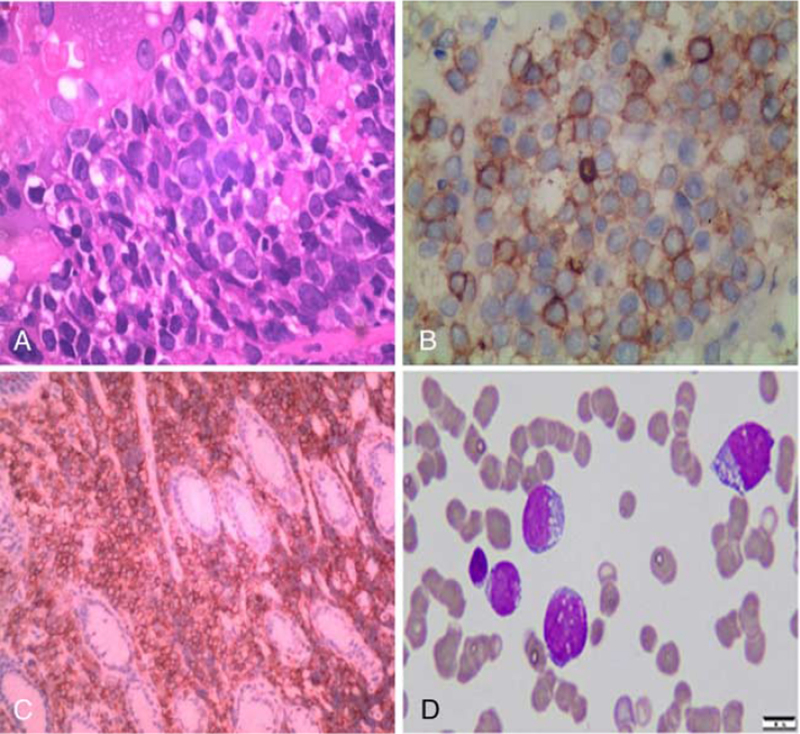
(A). H&E staining showing obvious atypical cell proliferation. IHC analysis showing the positive expression of CD117 (B) and MPO (C). (D). Bone marrow cytological examination showing active nuclear cell proliferation and myeloblast cells ≥88%. Note: (A–C) magnifications ×1000; (D) magnifications ×1000.

A 33-year-old woman who was suffering from persistent epigastric pain accompanied by anorexia and nausea without apparent inducements was admitted to our hospital in February 2018. Gastroscopy examination revealed a multiple cystic mass located in the gastric submucosa and the pathological diagnosis upon admission was chronic inflammation with lymph node hyperplasia. No further immunohistochemical labeling analyses were conducted and there was no obvious improvement of epigastric pain after receiving supportive treatment methods, such as antacids. On April 7, 2018, she was once again admitted to our hospital due to upper abdomen pain, anorexia, aggravated melena (100–200 g per time), and malaise. This patient had lost 10 kg since the onset of epigastric pain and had no personal or genetic family history of the disease. Examination on admission showed stable physical signs, chronic disease face, upper abdominal tenderness without superficial lymphadenopathy, rebound tenderness, muscle tension, double limbs edema, and splenomegaly. Moreover, no palpable mass was found in the abdomen through physical examination. Blood routine examination on admission showed the total amount of white blood cells (WBC) and red blood cells (RBC) to be 10.25 × 10^9^/L and 4.5 × 10^12^/L, while hemoglobin (Hb) and platelet (PLT) levels were 95 g/L and 618 × 10^9^/L, respectively. Gastroscopic examination revealed large stomach and duodenum mucosal fold (Fig. 1B–F) and multiple nodular projections (Fig. 1C and D). In addition, an approximately 1.8 cm × 1 cm ulcer was visible at the gastric angle (Fig. 1E), while no apparent abnormalities of esophageal morphology was observed (Fig. 1A). Bone marrow cytological examination showed obvious toxic changes in myeloid cells, while myeloblast cells were not observed (Fig. 2A). Moreover, immunohistochemical staining exhibited positive staining for Bcl-2 (+), cMyc (+), ki-67 (60%), CD117 (+) (Fig. 2B), CD34 (+), CD56 (+), MPO (+) (Fig. 2C), and CD79a (+). Based on World Health Organization (WHO) 2016 guidelines, this patient was diagnosed with isolated gastric MS and was considered for inducing chemotherapy (DA regimen: daunorubicin, mg/m^2^ d1-3; cytarabine, 150 mg/m^2^ d1-7). Considering the cachexia status of this patient, the family demanded that only palliative care, including nutrition support, antacids, blood transfusion, anti-infection, be administered.

**Figure 3:**
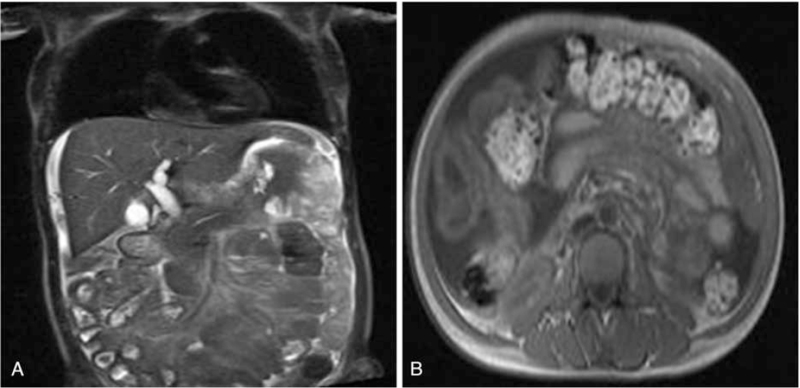
Whole abdomen MRI images showing extensive peritoneal thickening, multiple bowel-wall thickening, and edema in upper and middle abdomen.

**Figure 4:**
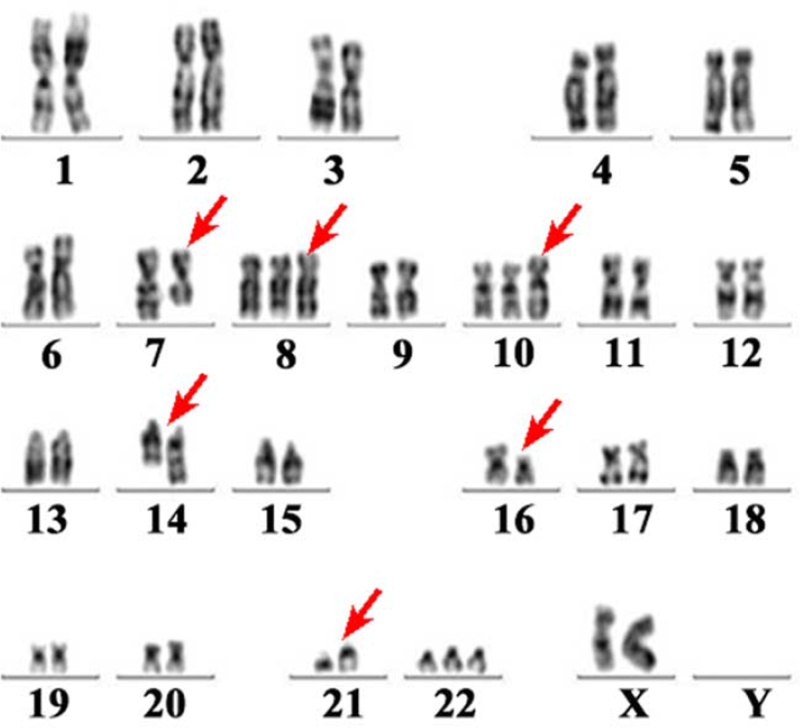
G-banding karyotype analysis of bone marrow showing complex karyotypic abnormalities (red arrow).

In August 2018, symptoms of abdominal distension gradually aggravated and were accompanied by large ecchymosis in total skin. Biochemical examination revealed WBC count of 124 × 10^9^/L, and PLT levels of 3 × 10^9^/L. The myelogram indicated active nuclear cell proliferation and 88% myeloblast cells (Fig. 2D). G-banded chromosome analysis of the patient's bone marrow cells revealed a composite complex karyotype of 46–50, XX, del (7) (q31) , del (7) (q32) , +8, +10, add (14) (q32) , t (16; 21) (p11; q22) , +22 [cp11]/48,X,del (X) (q24) , add (7) (p22) , del (7) (q32) , +10, add (14) (q32) , t (16; 21) (p11; q22) , +22, inc [5]/46. XX [4]. A total of 43 leukemia fusion genes were screened for using RT-qPCR and the results revealed positive TLS-ERG gene fusion. Meanwhile, whole abdomen MRI images revealed extensive peritoneal thickening and multiple bowel-wall thickening and edema in the upper and middle abdomen (Fig. 3). Therefore, we considered that isolated gastric MS patient had transformed into acute myeloid leukemia (AML) accompanied by TLS/ERG gene fusion and complex karyotypic abnormalities (Fig. 4). Similarly, the patient's family persistently requested that conservative and supportive treatment to be administered to this patient. Unfortunately, routine follow-up results demonstrated that this patient had died from cerebral hemorrhage in October, 2018. Ethics approval and documentation for the case report was authorized by the Ethics Committee of the Affiliated Hospital of Zunyi Medical University and written informed consent was obtained from patient's family.

## 3 Discussion

Given the fact that MS is a rare extramedullary myeloid tumor without any blood or bone marrow involvement at the time of diagnosis, it has been estimated that MS accounts for about 2%–9% of all AML cases, while the actual incidence of isolated MS is still relatively elusive.^[5]^ Several studies have reported that gastric MS can appear following the diagnosis of hematological neoplasms, isolated gastric MS is an exceedingly rare diagnosis.^[6,7]^ To the best of our knowledge, only 3 isolated gastric MS cases have been reported by other research teams.^[8–10]^ Hence, our current study may report on another isolated gastric MS case and provide novel evidence for the correct diagnosis of this extremely rare disease.

Considering the rarity and lack of typical clinical presentations of isolated gastric MS, it has been estimated that approximately 47% of cases are initially misdiagnosed and lymphoma is the most common misdiagnosis.^[11]^ Currently, histopathological diagnosis methods are still the gold standard for the diagnosis of MS. The differential diagnosis of MS from other malignancies, such as non-Hodgkin's lymphoma, Ewing's sarcoma, lymphoblastic leukemia, and extramedullary hematopoiesis is still done under a light microscope.^[12]^ Consistent with existing literature, this isolated gastric MS case was initially misdiagnosed as chronic inflammation with lymph node hyperplasia at a local hospital due to the lack of necessary IHC examination. This patient was advised to receive supportive treatment, including antacids, but abdominal pain showed no improvement. Indeed, the incorrect diagnosis of MS directly resulted in delaying treatment and reduced the life quality of the patient. Therefore, there is a definite need for the comprehensive integration of patient history with other diagnostic methods, including cytogenetic studies, molecular and genetic mutation analysis, mass and bone marrow biopsy, and imaging features, to correctly diagnose MS.

In addition, some myeloid-associated antigens, including MPO, lysozyme, CD68, CD43, CD34, CD99, and CD117 are frequently used to identify MS. Among these antigens, MPO exhibits the highest level of sensitivity and specificity. Immunohistochemically, the cells were positive for Bcl-2 (+), cMyc (+), ki-67 (60%), CD117 (+), CD34 (+), CD56 (+), MPO (+), and CD79a (+). Accordingly, the preliminary diagnosis of this patient was MS and further diagnostic studies should have been performed for clinical classification. Additionally, numerous reports have confirmed that MS can involve various extramedullary organs, including the skin lymph nodes, bone, brain, breast, cervix, and visceral organs.^[10]^ The clinical manifestations of MS depend on the primary tumor site. The initial symptom of this case was abdominal pain and whole abdomen MRI images revealed extensive peritoneal thickening, multiple bowel-wall thickening and edema in upper and middle abdomen. The pathological results suggested MS, while no immature cell infiltration was observed. This patient was thereby diagnosed with isolated gastric MS.

The TLS-ERG fusion gene was first identified in myxoid liposarcoma in 1993 and was derived from the translocation, t(16; 21) (p11; q22).^[13,14]^ Existing evidence has revealed that the TLS-ERG fusion gene is frequently observed in AML, blast crisis of chronic myelocytic leukemia, and Ewing's sarcoma.^[15]^ A comprehensive review of almost 50 ears of literature showed that less than 100 patients with TLS-ERG fusion gene have been documented.^[16]^ Up to now, there have been no clinical literature published on MS accompanied by TLS-ERG fusion gene. AML with TLS-ERG fusion gene is estimated to account for nearly 1% of all AML cases.^[17]^ It has been widely recognized that TLS-ERG fusion protein exerts its oncogenic role during the progression of AML is positively correlated with a poor prognosis and high risk of gene mutations, such as complex chromosome abnormalities and hematological malignancies gene mutations.^[16]^ Unfortunately, no fusion genes, cytogenetic studies, or molecular and genetic mutation analyses were performed when the patients receive the first diagnosis of isolated gastric MC. The common chromosome translocations in MS patients include t (8; 21), t (9; 11), del (16q), t (8; 17), t (8; 16), and t (1; 11).^[18–20]^ In our study, karyotype analysis of this patient showed complex chromosome abnormalities, including 46–50, XX, del (7) (q31), del (7) (q32), t (16; 21) (p11; q22), +22 [cp11]/48, X, del (X) (q24), add (7) (p22), del (7) (q32), +10, add (14) (q32), t (16; 21) (p11; q22), +22, inc [5]/46, XX [4]. A previous report by Tong et al.^[21]^ confirmed that in AML patients with TLS-ERG fusion gene, the disease was closely associated with extramedullary organs, complex chromosome abnormalities and a high-risk of gene mutation, which may partially contribute to the poor prognosis of TLS-ERG AML patients. This case of isolated gastric MS transformed into AML with a complex karyotype and TLS-ERG fusion gene, 4 months later. Consistently, the poor prognosis of this case might be partially ascribed to the occurrence of the TLS-ERG fusion gene.

To date, there has not been a consensus on the standard therapeutic regimen for MS in clinic practice due to the rarity of the disease. Currently used methods of treatment mainly include surgical resection, radiotherapy, systemic chemotherapy, targeted therapy, and hematopoietic stem cell transplantation (HCT).^[1]^ AML patients usually receive chemotherapy and consolidation chemotherapies, such as HD-Ara-C, TA regimen (pirarubicin and Ara-C), and EA regimen (VP16 and Ara-C), or HCT after complete remission.^[21,22]^ It is worth noting that most isolated MS patients eventually develop frank leukemia after receiving induction chemotherapy although long-term follow-up studies have demonstrated cases without progression.^[23]^ The previous comparison analysis showed that hematopoietic stem cell transplantation (HCT) can provide several benefits to prolong the overall survival (OS) of TLS-ERG^+^ AML patients (non-HCT vs HCT, 1-year OS: 62.5% vs 90%, *P* = .026; median OS time: 16 months vs 16 months), while no significant improvement of prognosis has been achieved.^[21]^ The patient in this study was advised to receive chemotherapy (DA regimen) when she was initially diagnosed with isolated gastric MS. However, only palliative care, including nutrition support, antacids, blood transfusion, anti-infection was administrated to this patient considering the cachexia status and the family's request. Then, isolated gastric MS rapidly transformed into AML, within 4 months. Finally, the interval time of this case from initial disease onset to death was nearly 5 months.

## 4 Conclusions

Isolated gastric MS is extremely rare and presents diagnostic and therapeutic challenges in clinical practice. Therefore, a comprehensive combination of patient history with imaging features, mass and bone marrow biopsy, and molecular cytogenetic studies may be crucial to avoid the misdiagnosis of isolated gastric MS. Furthermore, isolated gastric MS can transform into AML even within a short period if no appropriate systemic or local therapies are administered.

## Acknowledgments

We thank the patient's family for their approval to publication.

## Author contributions

**Conceptualization:** Lu Gao, Mingqiang Ren.

**Data curation:** Yan Xu, Junxiu Xia, Mingqiang Ren.

**Formal analysis:** Zuguo Tian, Di Chen.

**Funding acquisition:** Lu Gao.

**Methodology:** Zhong Yuan

**Writing – original draft:** Lu Gao, Mingqiang Ren.

**Writing – review & editing:** Lu Gao, Mingqiang Ren.
